# Supplementary figures and images for: Shedding of infectious SARS-CoV-2 despite vaccination
Source: PLoS Pathog. 2022 Sep 30;18(9):e1010876. doi: 10.1371/journal.ppat.1010876 (PMC9555632; doi:10.1371/journal.ppat.1010876)

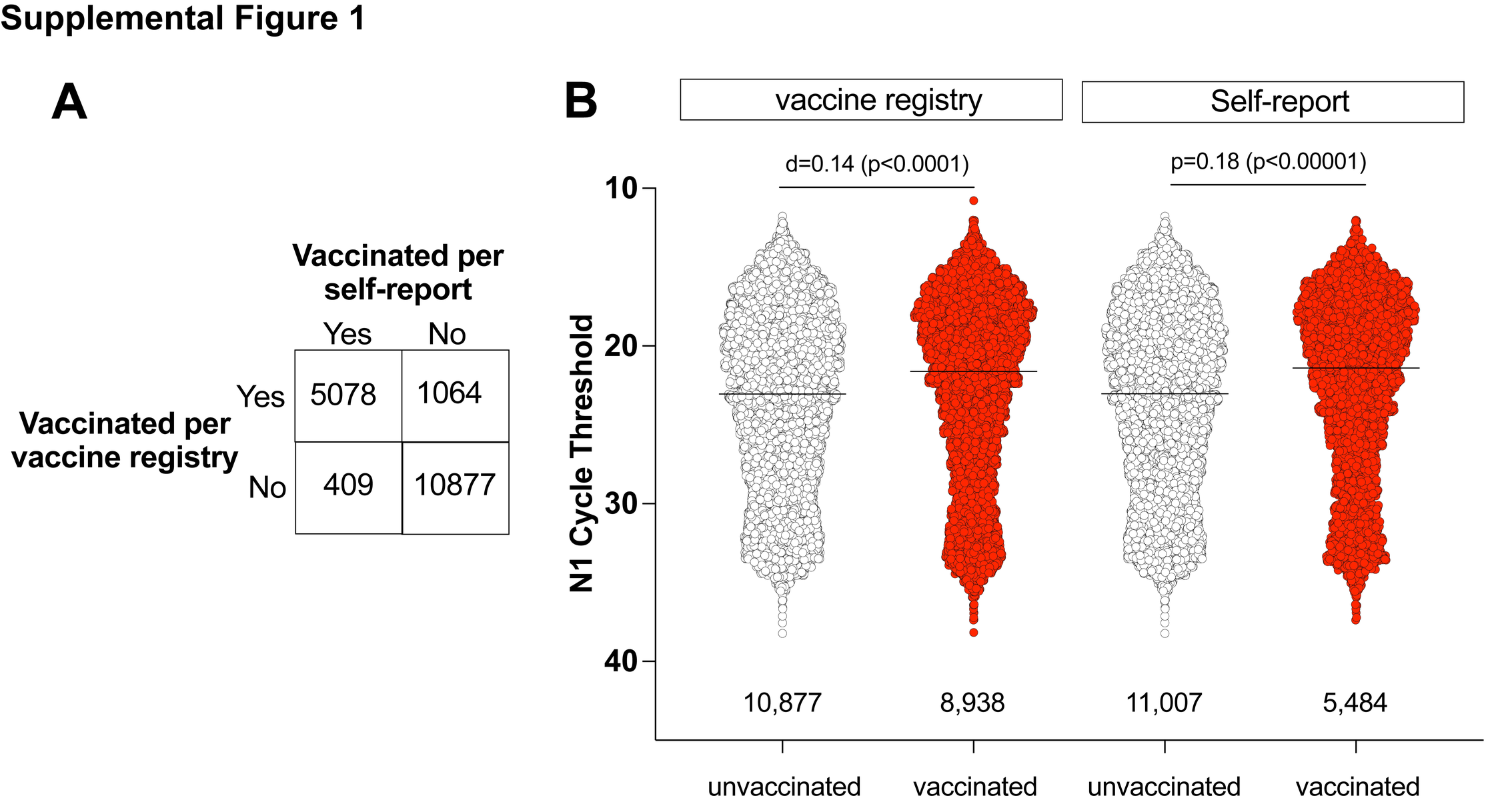

Supplement: S1 Fig — Individuals were considered fully vaccinated based on vaccine registry (WIR/WEDSS) data if the registries indicated receipt of a final vaccine dose at least 14 days prior to submitting the sample used in our analysis. For individuals whose vaccination status could not be verified in the registry, self-reported data collected at the time of testing were used. Individuals were considered unvaccinated based on self-report only if there was an explicit declaration of unvaccinated status in the self-reported data. Individuals were considered fully vaccinated based on self-report if they fulfilled all of the following criteria: (1) indicated that they had received a COVID vaccine prior to testing; (2) indicated that they did not require another vaccine dose; and (3) reported a date of last vaccine dose that was at least 14 days prior to testing. Specimens lacking data on vaccination status were excluded from the study. Specimens from partially vaccinated individuals (incomplete vaccine series, or <14 days post-final dose) were also excluded. Specimens from individuals who received a booster prior to sample collection were also excluded as non-equivalent to those fulfilling the criteria to be considered fully vaccinated. A. Of 20,431 specimens with vaccination status available from at least one source, 5,078 specimens had data available from both sources. Under-reporting of full vaccination status in self-reports 1,064/6,142 or 17%) was more common than over-reporting (409/5,487 or 7.4%). B. N1 Ct values for SARS-CoV-2-positive specimens grouped by vaccination status for individuals whose vaccination status was determined by vaccine registry or by self-reported data. (TIF) [file ppat.1010876.s001.tif]

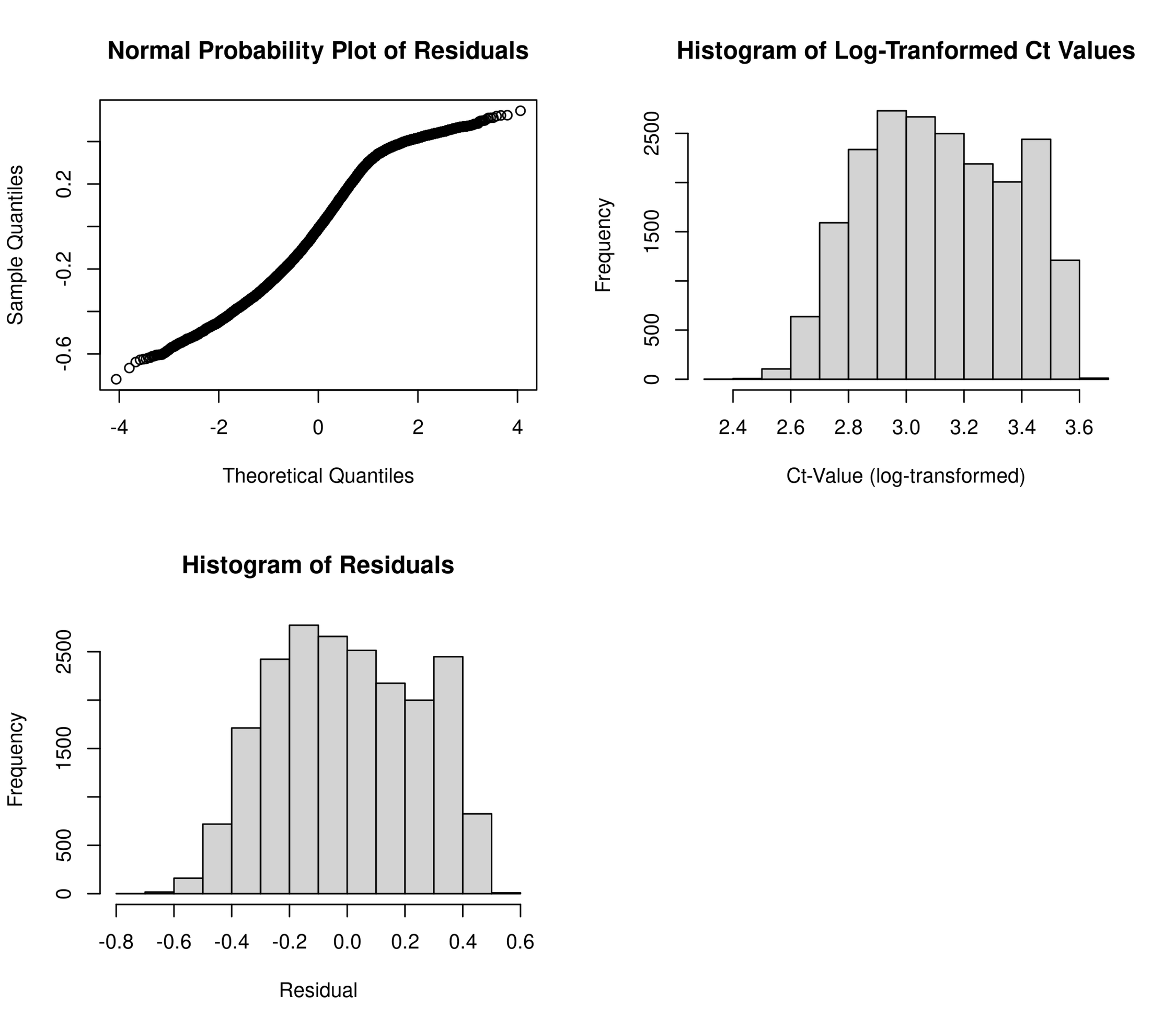

Supplement: S2 Fig — Raw Ct values were not normally distributed, so we log-transformed all Ct values prior to ANOVA, and confirmed normality by plotting residuals and normal probability. (TIF) [file ppat.1010876.s002.tif]

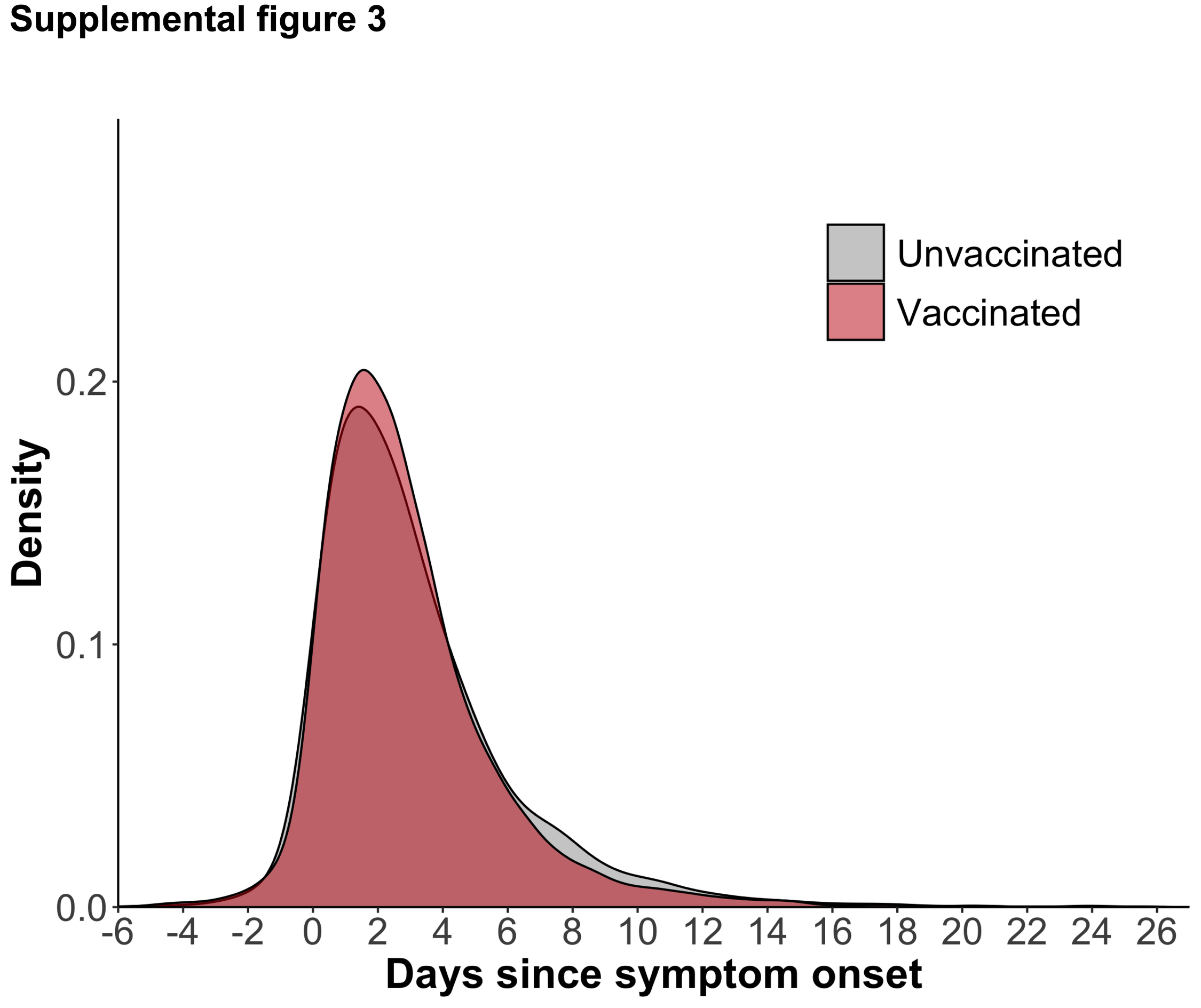

Supplement: S3 Fig — Day 0 on the x-axis denotes self-reported day of symptom onset. Negative values for days indicate specimen collection prior to symptom onset. Symptom onset data were available for n = 6,871 unvaccinated cases and n = 5,522 vaccinated cases. Two-sided K-S test: p = 0.0012; median days since symptom onset were 2.4 for both unvaccinated and vaccinated cases. (TIF) [file ppat.1010876.s003.tif]

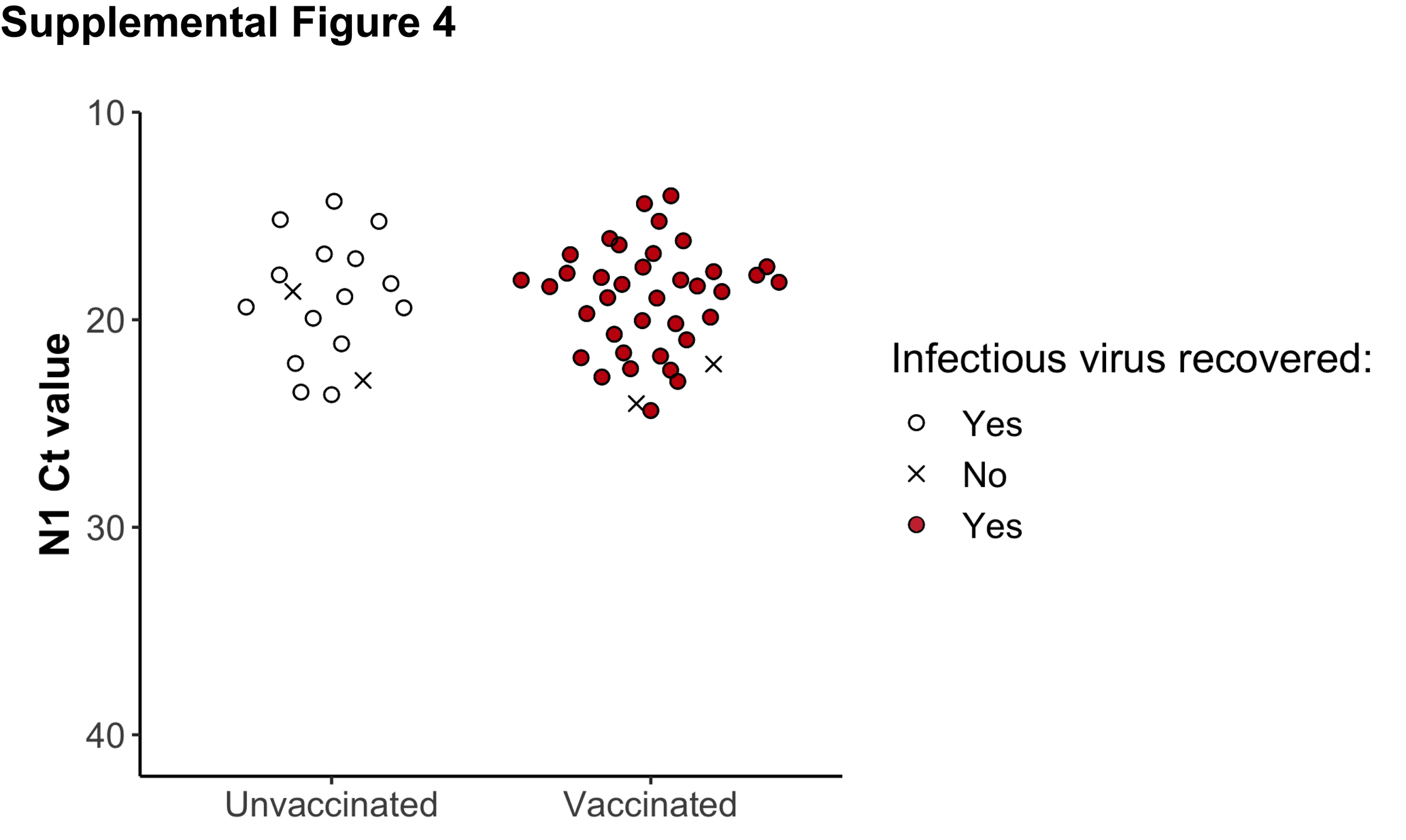

Supplement: S4 Fig — Infectiousness was determined for a subset of N1 Ct-matched specimens with Ct <25 by inoculation onto Vero E6 TMPRSS2 cells, then determining presence or absence of cytopathic effects (CPE) after 5 days in culture. Specimens with unknown vaccination status were excluded from the analysis. Circles indicate presence of CPE; ‘X’ indicates no CPE detected. (TIF) [file ppat.1010876.s004.tif]

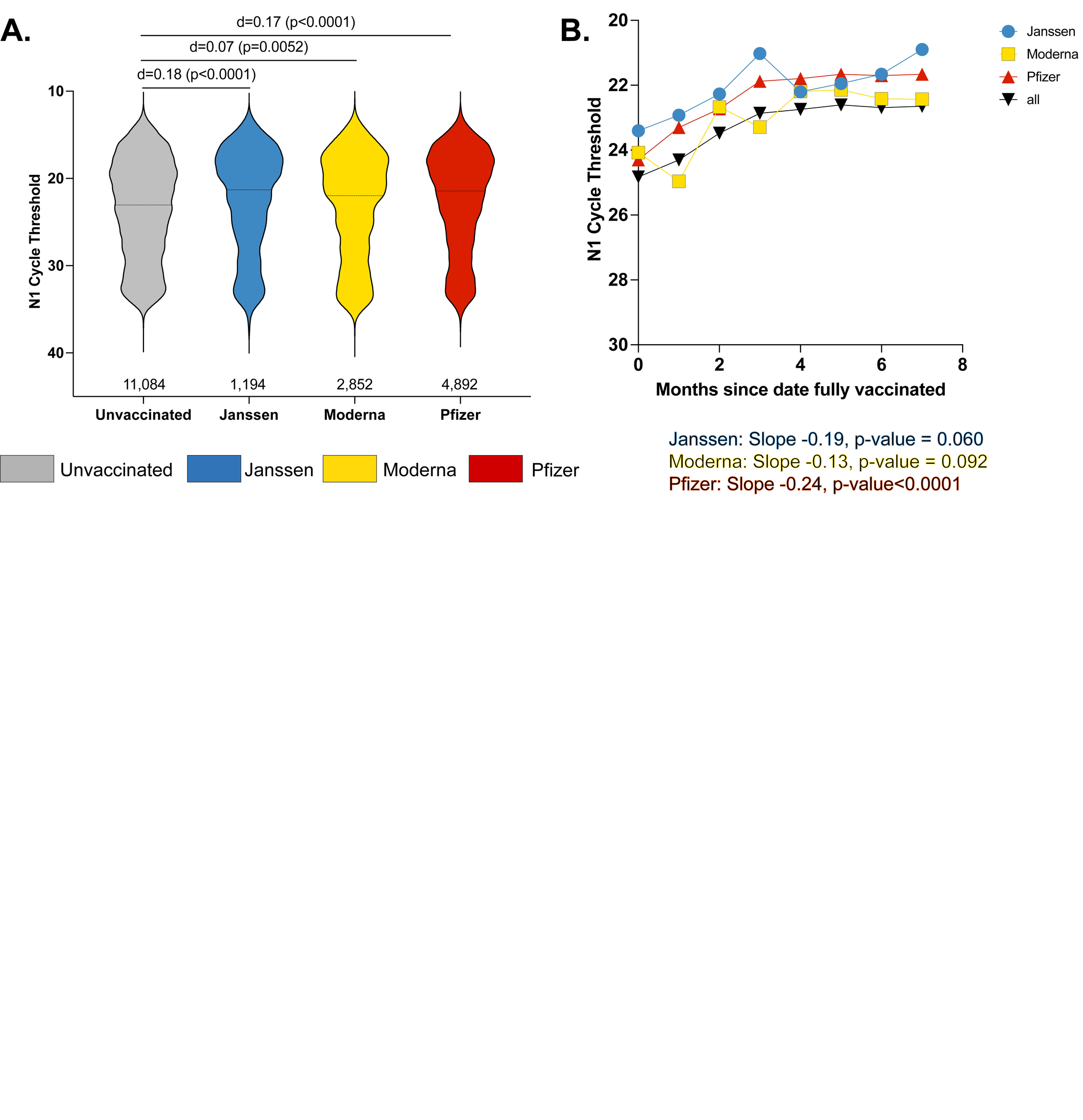

Supplement: S5 Fig — A. Comparison of mean N1 Ct values in all specimens, stratified by vaccine type shows negligible effect (d < 0.2) of vaccine type on Ct value at time of positive test, relative to unvaccinated persons. B. The time analysis showed a decrease in N1 Ct values with time over 7 months. Combining all three vaccines, there was a significant decrease over the first 7 months, with a slope of -0.18 (95% CI: -0.26–0.10), p value <0.0001. Individually, Janssen had a slope -0.19 (95% CI: -0.38 to -0.001, p-value = 0.060), Moderna had a slope of -0.13 (95% CI: -0.28–0.02, p-value = 0.092), Pfizer had a slope of -0.24 (95% CI: -0.24 to -0.13, p-value<0.0001). (TIF) [file ppat.1010876.s005.tif]
