# Supplementary material for: Shedding of infectious SARS-CoV-2 despite vaccination
Source: PLoS Pathog. 2022 Sep 30;18(9):e1010876. doi: 10.1371/journal.ppat.1010876 (PMC9555632; doi:10.1371/journal.ppat.1010876)
Supplement: S1 Table — Vaccination had negligible effects on mean Cts in vaccinated as compared with unvaccinated individuals, regardless of the vaccine manufacturer. (DOCX) [file ppat.1010876.s006.docx]

**Supplemental Table 1**: *Comparisons between vaccine type*

| **p- p- p- p- p- p- p-**  **Mean 95% CI** | | | | | | | | | |
| --- | --- | --- | --- | --- | --- | --- | --- | --- | --- |
|  |  |  | **value±** | **value1** | **value2** | **value3** | **value4** | **value5** | **value6** |
| Unvaccinated | 22.9 | 22.8-23.0 | <0.0001 | <0.0001 | 0.0052 | <0.0001 | 0.0064 | 0.9870 | 0.0001 |
| Janssen | 21.9 | 21.6-22.2 |  | | | | | | |
| Moderna | 22.5 | 22.3-22.7 |  |  |  |  |  |  |  |
| Pfizer | 22.0 | 21.8-22.1 |  |  |  |  |  |  |  |

‡ comparisons between all groups

**^1^**: comparison Unvaccinated vs. Janssen (adjusted for multiple comparisons using Tukey’s HSD method)

**^2^**: comparison Unvaccinated vs. Moderna (adjusted for multiple comparisons using Tukey’s HSD method)

**^3^**: comparison Unvaccinated vs. Pfizer (adjusted for multiple comparisons using Tukey’s HSD method)

**^4^**: comparison Janssen vs. Moderna (adjusted for multiple comparisons using Tukey’s HSD method)

**^5^**: comparison Janssen vs. Pfizer (adjusted for multiple comparisons using Tukey’s HSD method)

**^6^**: comparison Moderna vs. Pfizer (adjusted for multiple comparisons using Tukey’s HSD method)
