# Supplementary material for: Shedding of infectious SARS-CoV-2 despite vaccination
Source: PLoS Pathog. 2022 Sep 30;18(9):e1010876. doi: 10.1371/journal.ppat.1010876 (PMC9555632; doi:10.1371/journal.ppat.1010876)
Supplement: S2 Table — Vaccination status had negligible effects on Ct values (d<0.2) for all age groups considered except those aged 0–11 years—there is a significant interaction between age group and vaccination status, p<0.0001. However, in this group, there were very few vaccinated individuals (N = 7), as would be expected because vaccines had not been approved for those 11 and under for most of our study period. Therefore, despite the significant effect size, we do not believe our data strongly support the notion that vaccination status has a strong effect on Ct value in children under 12. (DOCX) [file ppat.1010876.s007.docx]

**Supplemental Table 2**: *Comparison of Ct values in vaccinated and unvaccinated persons, stratified by age group (there is a significant interaction between age group and vaccination status, p<0.0001)*

|  | **Not Vaccinated**  **Mean 95% CI** | | **Vacci**  **Mean** | **inated**  **95% CI** | **Effect size *d*** | **p-value** |
| --- | --- | --- | --- | --- | --- | --- |
| 0-11 yr | 23.9 | 23.7-24.1 | 19.8 | 16.5-23.8 | 0.79 | 0.0466 |
| 12-18 yr | 23.0 | 22.8-23.3 | 23.9 | 22.5-23.5 | 0.00 | 0.9242 |
| 19-35 yr | 22.4 | 22.2-22.6 | 23.0 | 22.1-22.6 | 0.00 | 0.8846 |
| 36-60 yr | 22.3 | 22.1-22.5 | 21.9 | 21.8-22.1 | 0.07 | 0.0080 |
| >61 yr | 22.3 | 21.9-22.8 | 22.1 | 21.8-22.3 | 0.05 | 0.3239 |
|  |  |  |  |  |  |  |
