# Supplementary material for: Shedding of infectious SARS-CoV-2 despite vaccination
Source: PLoS Pathog. 2022 Sep 30;18(9):e1010876. doi: 10.1371/journal.ppat.1010876 (PMC9555632; doi:10.1371/journal.ppat.1010876)
Supplement: S3 Table — When comparing Ct values between unvaccinated and vaccinated within males and females, negligible differences were observed. (DOCX) [file ppat.1010876.s008.docx]

**Supplemental Table 3**: *Comparison of Ct values in vaccinated and unvaccinated persons, stratified by sex.*

|  | **Unvaccinated** | **Vaccinated** |  | |
| --- | --- | --- | --- | --- |
|  | **Mean 95% CI** | **Mean 95% CI** | **Effect size *d*** | **p-value** |
| Female | 23.0 22.9-23.2 | 22.3 22.1-22.4 | 0.14 | <0.0001 |
| Male | 22.8 22.6-22.9 | 22.0 21.8-22.1 | 0.15 | <0.0001 |
|  |  |  |  |  |
