# Supplementary material for: Shedding of infectious SARS-CoV-2 despite vaccination
Source: PLoS Pathog. 2022 Sep 30;18(9):e1010876. doi: 10.1371/journal.ppat.1010876 (PMC9555632; doi:10.1371/journal.ppat.1010876)
Supplement: Attachment — Submitted filename: 220624 Response to Reviewers.pdf [file ppat.1010876.s009.pdf]

WISCONSIN
UNIVERSITY OF WISCONSIN–MADISON

Nancy Wilson, PhD
AVRL
555 Science Drive
Madison, WI 53711

June 24, 2022

To the PLoS Pathogens editors and reviewers:

Thank you very much for your perceptive reviews of our manuscript PPATHOGENS-D-21-02286, “Shedding of Infectious SARS-CoV-2 Despite Vaccination.” We are submitting a significantly revised manuscript, which we believe is much improved by the reviewers’ suggestions. We have made changes to the text throughout to update it and provide additional context, since substantial time has passed since the initial submission. We also would like to thank you for the extension granted in returning this revised manuscript.

A specific concern raised by reviewer #1 was that substantial additional metadata, such as vaccine product received, time since vaccination, and age and sex of individuals testing positive, were needed to make this manuscript publishable. In the original submission, we did not include these data, due in large part to the difficulty in accessing such information from public health systems. To address this concern, we negotiated a new Data Use Agreement with the State of Wisconsin to enable sharing of these data. Such agreements are time-consuming to develop and for the State to review, largely accounting for the delay in this resubmission.

Nonetheless, this delay also provided the opportunity for us to address other reviewers' concerns by greatly expanding the dataset. In the previous submission, the data encompassed the time period 6/28/21 - 7/31/21. In this revised version, the data cover a longer time period, beginning when the Delta VOC became predominant in Wisconsin, 6/28/21, and ending 12/1/21, just prior to the first detection of an Omicron variant in the state. With the increased sample size (20,341), as well as additional metadata for the samples obtained from Wisconsin Department of Health, we believe we were fully able to address the reviewers’ concerns.

In the following paragraphs, we present a point-by-point response to the concerns presented by the reviewers. We responded to as many reviewer concerns as possible in the manuscript text, but in some cases we felt that reviewer concerns addressed questions that are scientifically interesting, but which our study was not designed to address with rigor. In such cases we have presented data that are responsive to reviewer concerns, but we have elected to leave these data out of the manuscript itself. Overall we feel that our manuscript is much improved and hopefully is now acceptable for publication.

Sincerely yours,

Nancy Wilson , PhD.

Point-by-point responses to the reviewers' comments (shown in italics) are listed below along with cross-references to locations in the manuscript where changes have been incorporated into the manuscript. If a comment was made more than once, only the first instance is addressed.

One advantage of our study is that we compared results from a single SARS-CoV-2 PCR assay conducted by a single large commercial testing provider, thereby minimizing bias that would result from comparisons of results form different laboratories conducting different test types. It is imporant to note, however, that our study was not specifically designed to determine whether vaccination (or specific vaccine products) reduced the level or duration of SARS-CoV-2 burdens. Rather, our main question was simply whether vaccinated individuals who became infected could plausibly contribute to SARS-CoV-2 transmission, as public health recommendations at the time of study initiation held that vaccinated individuals might be exempt from some mitigation measures, such as indoor masking, where unvaccinated individuals should still follow them.

To add rigor to our statistical analyses, and to perform the additional analyses requested by Reviewer #1, we collaborated with Dr Jens Eickhoff, a biostatistician. Dr Eickhoff led the expanded analyses we present below, and summarized his approaches in a new subsection in Methods. As a result of these analyses, we have added 1 new table to the body of the text and 3 tables to the Supplemental Methods; these will be discussed in more detail below.

Lines: 108 - 133
Table 1
Supplemental Tables 1-3

Reviewer #1:

The authors do not differentiate between patients which have received different vaccines, nor do they differentiate swabs based on the precise time after vaccination in which these individuals have tested positive. Given the published differences in protection which are elicited from the different vaccines (RNA vaccines vs Adv5 vaccines for example), it is difficult to compare between these groups and form conclusions on the ability of delta to infect vaccinated individuals

The reviewer is correct that there is the possibility that different vaccine products could provide different levels of protection against high viral burdens in breakthrough infection. We again note that our study was not designed prospectively to detect effects of vaccine product on Ct value after breakthrough infection, but our expanded dataset does allow us to stratify by product. In the revised Supplemental Figure 5A, we compare Ct values in specimens from unvaccinated persons to those obtained from individuals who received different vaccine products. We saw no difference in Ct value in vaccinated persons according to vaccine product received.

To determine whether time between last vaccine dose and positive test affected Ct values in vaccinated individuals, we stratified Ct values in vaccinated persons by month since last vaccine dose. We found a small, but significant, decrease in Ct value with time between vaccination and infection. We also stratified these data by vaccine product. The results, shown in Supplemental Figure 5B, indicate that the relationship between Ct value and time

since vaccination is significant only for individuals receiving the Pfizer vaccine. This effect appears to be driven largely by a relatively high mean Ct value in Pfizer recipients who tested positive within 15-30 days of vaccine receipt. In sum, these additional analyses support our overall conclusion that breakthrough infection with Delta viruses can result in low Ct values indicative of high viral burdens in many vaccinated individuals, regardless of vaccine product received, and even if the interval between vaccination and infection is short.

Lines: 167 - 205
Supplemental Figure 5
Supplemental Table 1

Furthermore, the methods provided by the authors lacks detail.

We have expanded the Methods section to include details about the RNA extraction and RT-PCR. A strength of this dataset is that all of the samples were extracted and PCR amplified using a single assay at a single commercial test provider.

Lines: 73 - 83

We have described in more detail how we parsed data into the vaccinated and unvaccinated populations and which data were included or excluded. The R script used to parse the data into vaccinated and unvaccinated categories will also be included in the shared data folder.

Lines: 84 - 98

We have added details describing how the plaque assays were carried out.

Lines: 99 - 107

Further confounding factors such as age and sex should also be investigated to determine if this biases the data.

We appreciate the reviewer's point - vaccine became available at different times according to age, and as one might expect, there are relatively few children, especially those aged 11 and under, among the vaccinated individuals in our data. If SARS-CoV-2 viral loads systematically differ in young children relative to older individuals, this could skew our comparisons of Ct values in vaccinated vs. unvaccinated individuals without correcting for age. Again, we note that our study was not prospectively designed to rigorously address whether factors such as age and sex affected Ct value in infected persons, but rather whether Ct values consistent with high viral burdens occur frequently in vaccinated persons with breakthrough infection.

Nonetheless, we agree that these analyses strengthen and contextualize our results, and we have therefore added Supplemental Tables 2 and 3 to the manuscript. We further present here figures to address age and sex as potential confounding factors.

Vaccination had negligible effects on Ct values at the time of testing when stratifying by age group in individuals aged 12 and over, as is now shown in Supplemental Table 2.

Vaccination was approved for children aged 5-11 only on 29 October 2021, about a month before the end of our study period. When comparing Ct values in children aged 0-11, we do find a moderate effect of vaccination status on Ct value, with lower mean Ct values among vaccinated children (n=7) than unvaccinated children (n=3,132; *d*=0.79; Reviewer Figure 1). However, the very low number of infected, vaccinated children aged 0-11 makes it difficult to draw strong conclusions from our data about the effect of vaccination on Ct values among children under 12.

We also stratified Ct values by sex. Male and female populations are similar in size, have similar means and the effect size is small. Thus, we find no evidence that sex affects Ct value at the time of testing.

Lines: 182 - 190

Supplemental Tables,2,3

Age as a potential confounding factor

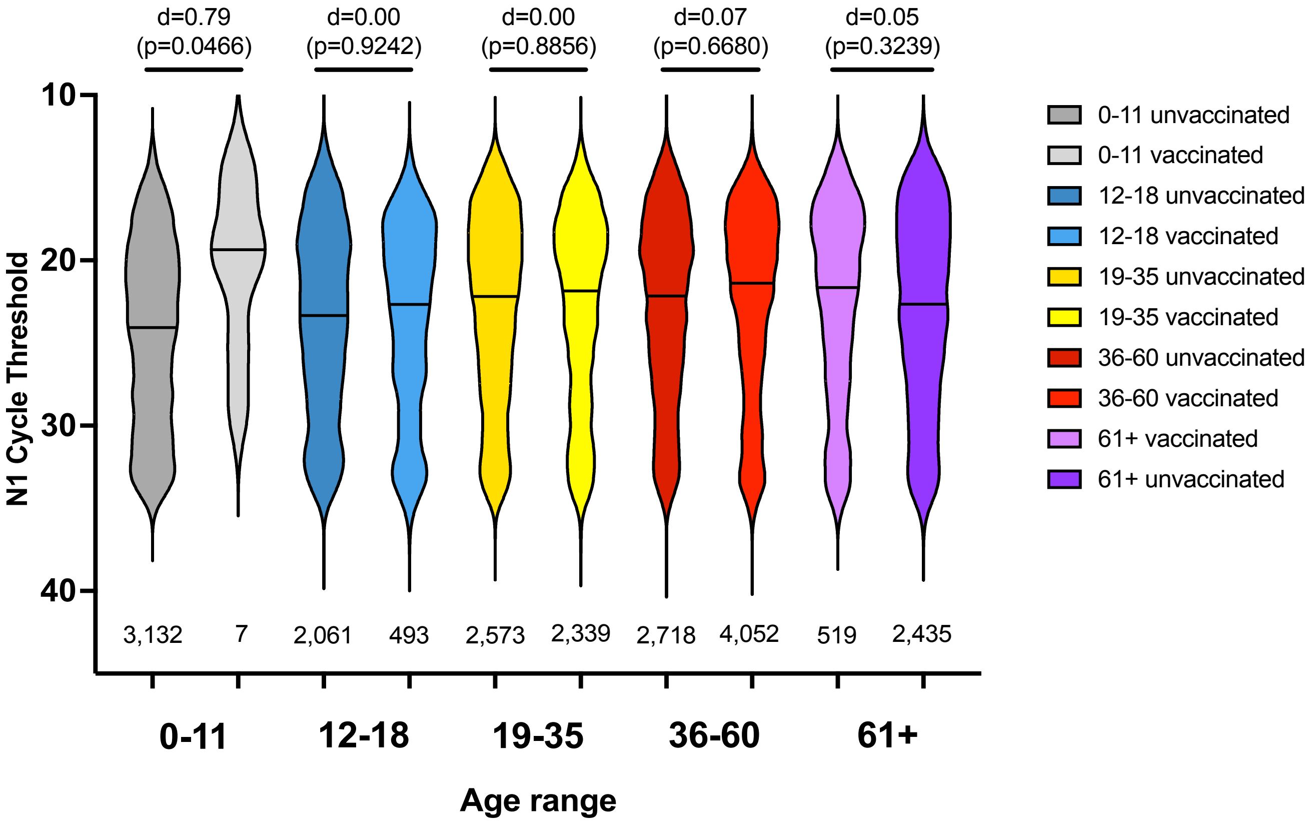

Rebuttal Figure 1. Age as a potential confounding factor. Ct values from vaccinated and unvaccinated individuals were stratified by age group. There is no significan interaction between age group and vaccination status. The number of individuals in each group is listed underneath the violin plot. Cohen's d values <0.2 are considered to be a negligible difference. The only significant difference is in the age group 0-11 and one can argue that this group is extremely biased due to the very small number of samples in the vaccinated group. See also Supplemental Table 2.

Sex as a potential confounding factor.

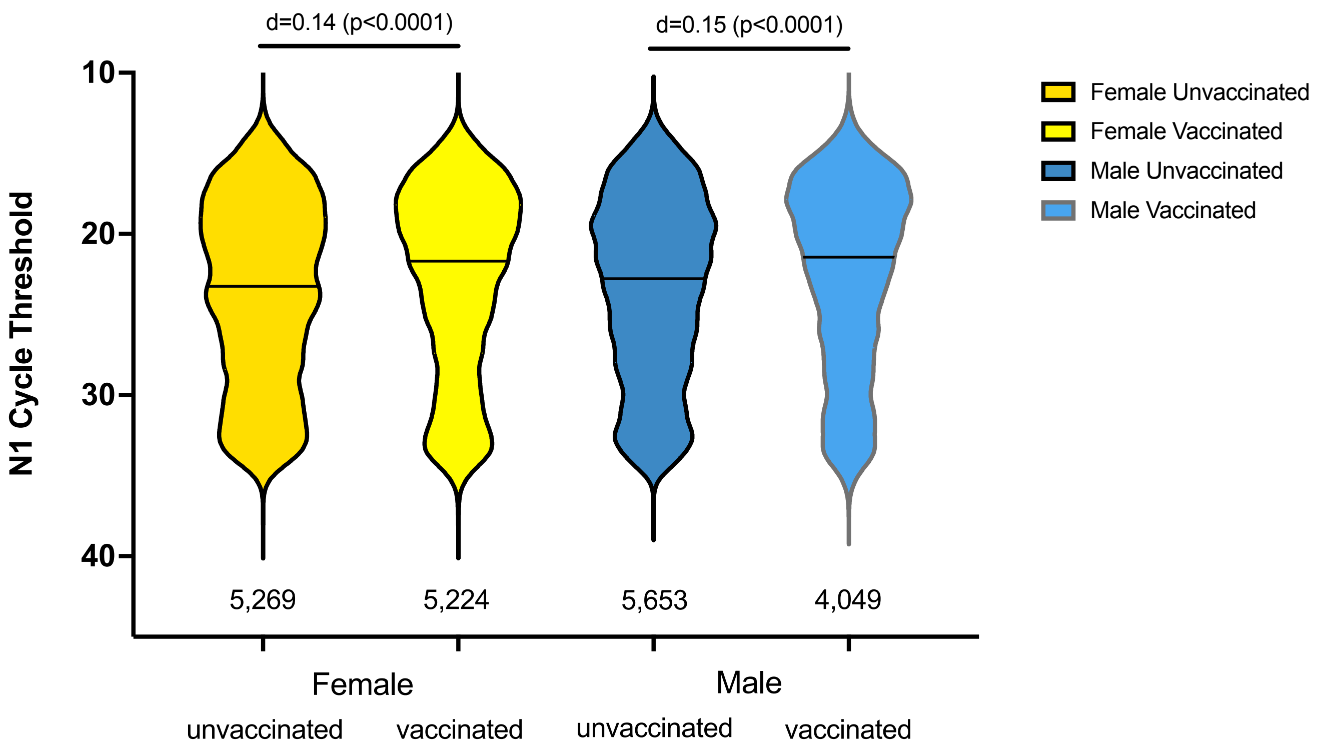

Rebuttal Figure 2. Sex as a potential confounding factor. Ct values from vaccinated and unvaccinated individuals were stratified by sex. Number of samples in each group are indicated below each violin. Effect sizes indicate that there are no differences between vaccinated or unvaccinated samples for either sex. See also Supplemental Table 3.
