# Supplementary material for: Shedding of infectious SARS-CoV-2 despite vaccination
Source: PLoS Pathog. 2022 Sep 30;18(9):e1010876. doi: 10.1371/journal.ppat.1010876 (PMC9555632; doi:10.1371/journal.ppat.1010876)
Supplement: Attachment — Submitted filename: 220818 Response to Reviewers.pdf [file ppat.1010876.s010.pdf]

WISCONSIN
UNIVERSITY OF WISCONSIN–MADISON
Nancy Wilson, PhD
AVRL
555 Science Drive
Madison, WI 53711

12 August 2022

To the PLoS Pathogens editors and reviewers:

Thank you very much for your thoughtful reviews of our manuscript PPATHOGENS-D-21-02286, “Shedding of Infectious SARS-CoV-2 Despite Vaccinations.” We have updated the study design methods section to better reflect STROBE guidelines for a cross-sectional study. In the following paragraphs, we present a point-by-point response to the concerns presented by the reviewers. Overall, we feel that our manuscript is much improved and hopefully is now acceptable for publication.

Sincerely yours,

Nancy Wilson, Ph.D.

Point-by-point responses to the reviewers' comments (shown in italics) are listed below, along with cross-references to locations in the manuscript where changes have been incorporated into the manuscript.

Reviewer #2: Overall the authors have done a very nice job responding to the reviewers concerns. In particular, it does not appear that age or sex or other examined variables introduced bias of concern into the study. However, the methods, in particular the study design area could still be clarified. The authors should consult STROBE guidelines for a cross-sectional study.

Thank you for the excellent suggestion. We have consulted the STROBE guidelines and adjusted the methods section to conform.

Lines: 74-86

Reviewer #2: To clarify the study design methods the authors should:
1) State clearly at the beginning of the study design section that this is a cross-sectional study.

We moved reference to a cross-sectional study from line 70 to the front of the section at line 64.

Lines: 75

2) Provide some detail to clarify the setting. That is, where the samples were collected and why. One to two sentences should suffice.

Description of why samples were collected was moved to the beginning of the Study Design section. A description of the study objective was also included in this section.

Lines: 75-76

Location of sample collection is described in lines 70-71.

Lines: 82-83

3) I recommend that the dates sentence be moved from the last sentence of the intro into the methods.

Reference to the dates of the study is now moved to line 76. It was removed from the end of the introduction and the end of the study design section.

Line: 76
4) Not use the word "cohort" in the study design section when they refer to the participants in their study.

The word "cohort" has been removed from the study design section.
Lines: 75-86
